# An Unusual Case of Inflammatory Pseudotumor of Paratesticular Region

**DOI:** 10.7759/cureus.46768

**Published:** 2023-10-09

**Authors:** Mohamed Javid, Sudhakaran Selvaraj, Ramesh Ganapathy, Senthikumar Sivalingam, Srikala Prasad

**Affiliations:** 1 Urology, Chengalpattu Medical College, Chengalpattu, IND

**Keywords:** inflammatory pseudotumor, paratesticular tumor, fibrous pseudotumor, scrotal mass, paratesticular region

## Abstract

Inflammatory pseudotumors (IPT) are rare benign tumors that can develop in various regions of the body. Notably, IPTs have been uncommonly described in the Genitourinary system including occasional reports from the paratesticular region. This origin from this area is significant because of the ambiguity in differentiating this pathology from malignant lesions arising from the testis. We would like to report a case of a 32-year-old male who presented with a painless left scrotal mass for two years. Ultrasonography was done followed by a radical orchidectomy and histopathological examination of the excised lesion showed features suggestive of inflammatory pseudotumor from the paratesticular region. We feel this case merits reporting owing to the rarity of IPT occurring in the paratesticular region and the essential need for increased awareness and differentiation from the malignant counterparts.

## Introduction

Inflammatory pseudotumors (IPT) are benign tumors that can originate from various organs in the body [1,2]. Though IPTs are commonly described in the lungs and the orbit, these tumors have also been reported in multiple extrapulmonary sites [3]. The first description of IPT was by Gleason and Busse in 1903 with further characterization by Birch-Hirchfeld in 1905 [4]. Later in 1954, Umiker and Iverson coined the term “inflammatory pseudotumor,” because of its aggressive clinical nature which resembles that of a malignant tumor [4]. Balloch et al. were among the earliest to report a lesion confined to the paratesticular region in 1904 [1,2]. Within the genitourinary system, it is frequently seen in the urinary bladder, often with a history of iatrogenic manipulation [5]. Genitourinary IPTs account for nearly 9% of extrapulmonary IPTs [2]. Furthermore, reports of paratesticular IPTs remain sporadic in contemporary literature, highlighting the significance of documenting such occurrences [2]. When located in the paratesticular region, these tumors commonly present as an incidental intrascrotal mass. This clinical presentation mimics that of malignant neoplasms, underlining the vigilant need for careful and thorough diagnostic evaluation [6]. The recommended definitive management generally is complete surgical excision [5].

## Case presentation

**Figure 1:**
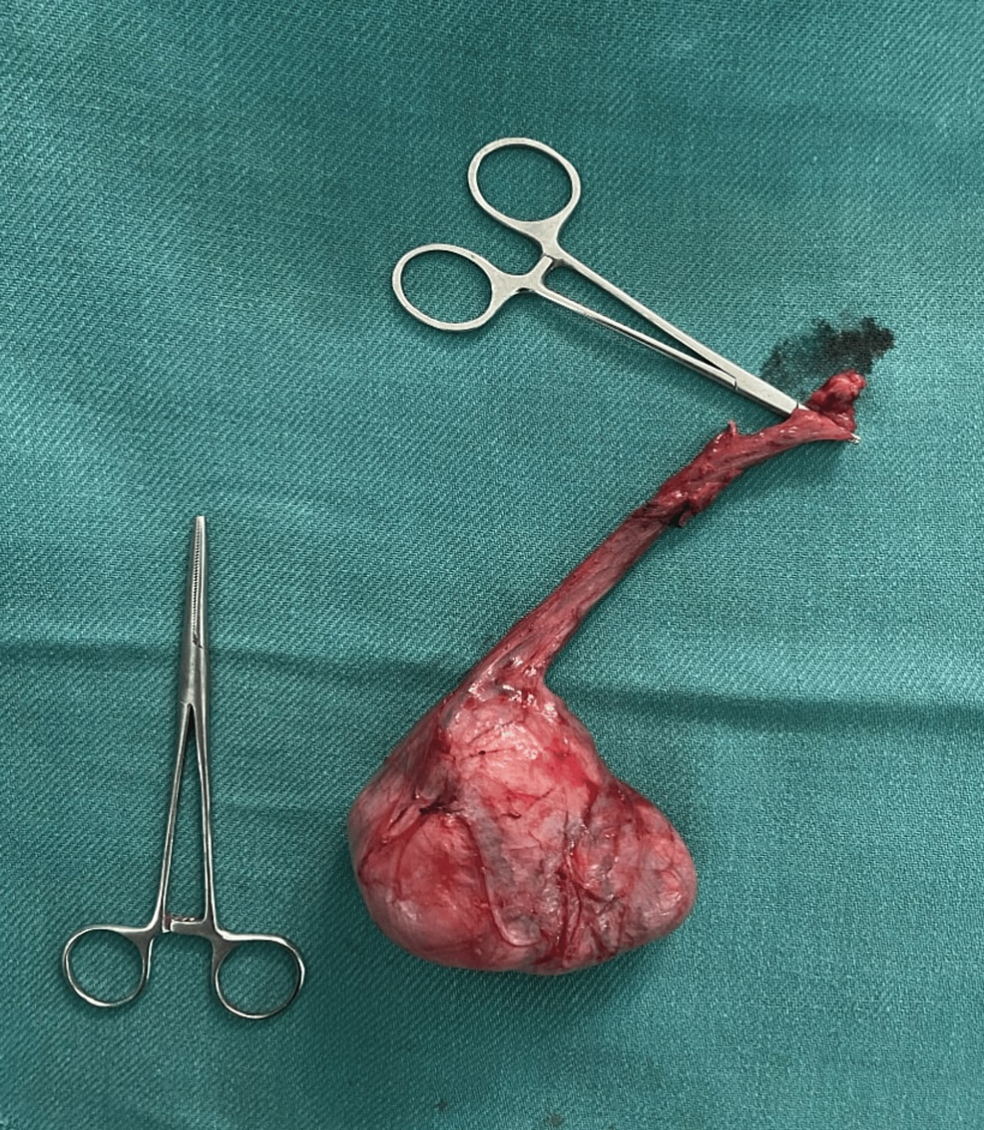
Tumor and testis specimen after radical orchidectomy.

A 32-year-old male patient presented to us with complaints of a painless left scrotal mass for two years. The scrotal mass was insidious in onset, progressive in nature, and hard in consistency with no history of any antecedent trauma or recurrent infections. Clinical examination revealed a hard, non-tender mass of size 7 x 5 x 3 cm in the left hemi-scrotum which could not be palpated separately from the testis. The mass was not adherent to the overlying scrotal skin and there were no other swellings in the groin or other regions of the body. Ultrasonography was done and this showed a heterogeneous mass of size 6 x 6.5 x 2.5 cm in the left scrotum. Serum tumor markers namely alpha-fetoprotein (AFP), beta human chorionic gonadotropin (hCG), and lactate dehydrogenase (LDH) were found to be within normal limits. This was followed by a radical orchidectomy and a gross examination of the specimen showed a 7.5 x 7 x 3.5cm vaguely nodular, capsulated mass attached to the testis and cord, which on cross-section was greyish white, firm, and whorled (Figure 1).

**Figure 2:**
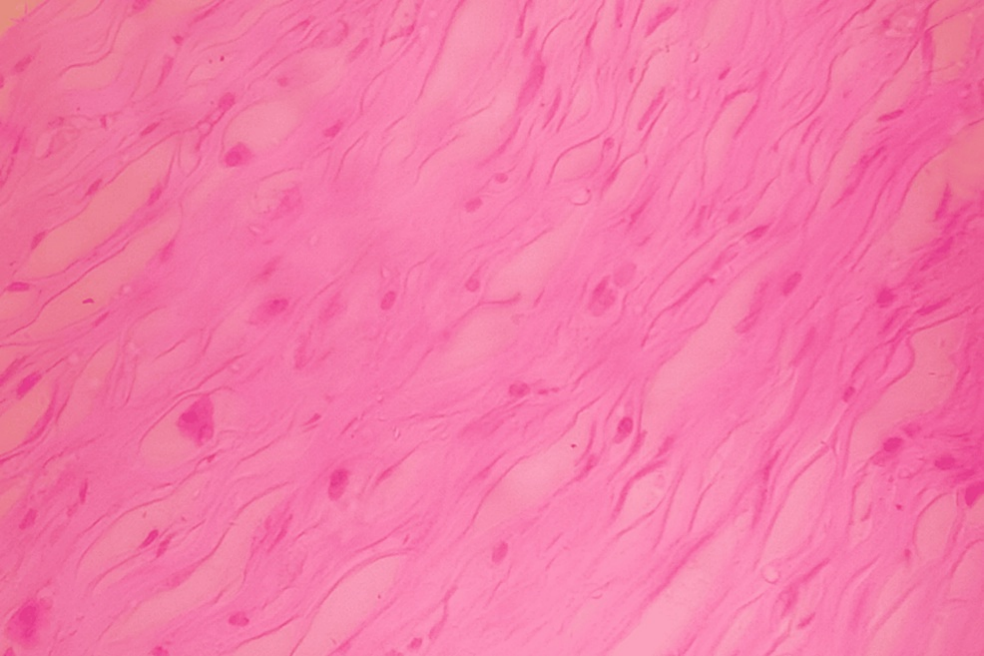
HPE demonstrating spindle-shaped cells with elongated slender nuclei with eosinophilic cytoplasm arranged in whorls and fascicles

The histopathological examination (HPE) of the specimen demonstrated spindle-shaped cells with elongated slender nuclei with eosinophilic cytoplasm arranged in whorls and fascicles (Figure 2). The testis and epididymis were found to be normal on HPE.

**Figure 3:**
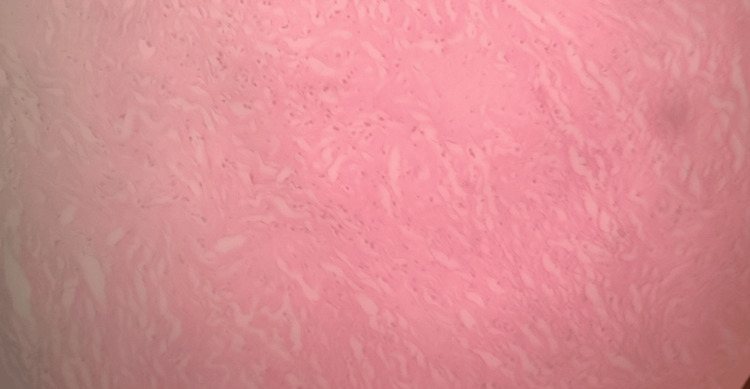
HPE demonstrating the intervening areas showing extensive hyalinization

**Figure 4:**
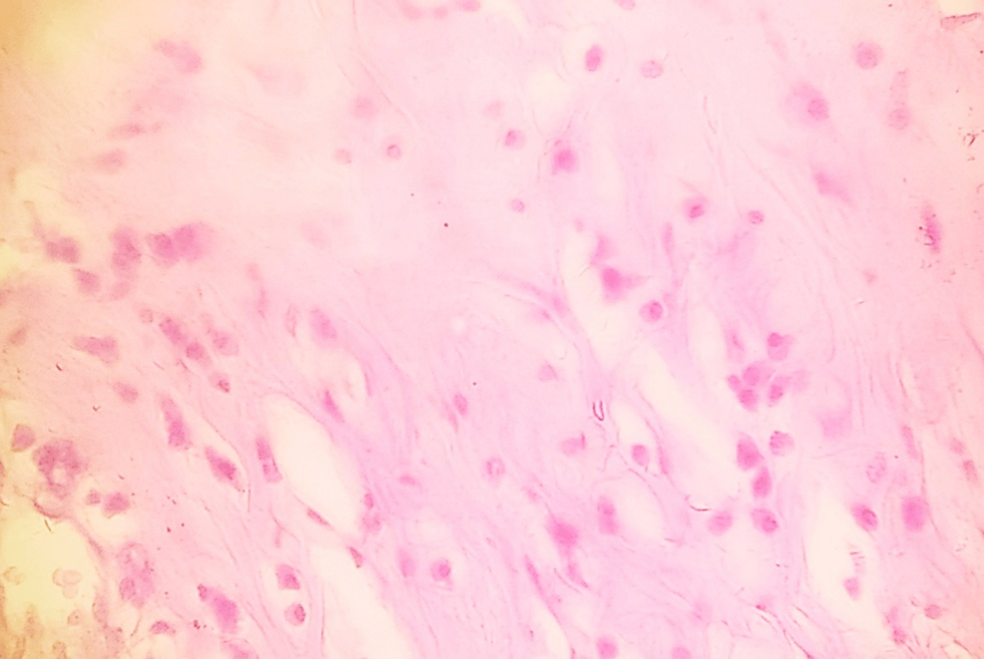
HPE demonstrating inflammatory cell infiltration

The intervening areas show extensive hyalinization and inflammatory cell infiltration composed of plasma cells, lymphocytes, and eosinophils (Figures 3, 4).

These features directed us to a conclusion of the Inflammatory Pseudotumor of the paratesticular region. The patient was followed up at six months and he was asymptomatic with no evidence of any recurrence.

## Discussion

Paratesticular tumors are lesions that constitute approximately 5% of the overall intrascrotal neoplasms. Among these, approximately 75% of these tumors arise from the spermatic cord [7]. Also, paratesticular IPT contributes to 6% of the overall paratesticular tumors and lesions [8]. In the paratesticular region, these growths may emanate from the tunics, epididymis, and spermatic cord [2]. Though these tumors are commonly seen in children and young adults, they can be seen in all age groups and there are even reports from the elderly population [2]. These tumors are frequently encountered within the pulmonary system in pediatric populations, and in the abdominal and retroperitoneal regions among adults [5,8]. Interestingly, the genitourinary tract is an uncommon location for this rare pathology. Within the genitourinary system, IPT occurrences have been noted in structures such as the kidneys, urethra, prostate gland, ureters, paratesticular area, and testes. Notably, the urinary bladder emerges as the predominant site of IPT occurrence within the genitourinary system [2]. They have been cited using various names in the literature such as inflammatory pseudotumor, plasma cell pseudotumor, xanthomatous pseudotumor, atypical myofibroblastic tumor, atypical fibromyxoid tumor, pseudosarcoma, plasma cell granuloma, and fibrous pseudotumor [9].

It is thought to be a consequence of spindle cell proliferation but the exact underlying etiopathogenesis has not been fully understood [5]. However, it has been hypothesized that chronic irritation, ischemia, and infection can be the underlying reason for the origin of this tumor [2]. Macroscopic appearance is usually a solid, nodular lesion with well-defined contours and a greyish-white cross-sectional area with diffuse growth patterns. Histological examination generally demonstrates spindle cell proliferation in loose collagenous tissue with mixed inflammatory cell infiltration [10]. The usual clinical presentation is a painless scrotal mass for a variable duration [11]. Ultrasonography stands as the initial investigative modality of choice. This tool not only aids in differentiating a testicular mass from a paratesticular mass but also provides insights into the characteristics of the lesion [8]. During the evaluation, it is imperative to exclude more common pathologies such as varicocele, spermatocele, infections, and malignant neoplasms. Also, as a basic principle, any firm intratesticular mass must be considered cancer until proven otherwise, and further examination with a scrotal ultrasound is crucial to ensure that malignancies are not overlooked [7]. However, the definitive differentiation of IPT from a malignancy can be done only on postoperative histopathological examination of the excised specimen.

The definitive management of IPT is the complete excision of the lesion [5]. Considering the fact that a significant portion of patients presenting with these tumors is young, a testicular-sparing approach with complete local excision of the tumor is to be contemplated whenever possible [12]. However, when there is a strong suspicion of malignancy or in situations where the mass cannot be separated clearly from the testis a radical orchidectomy is justified. Adjuvant chemotherapy or radiotherapy is not recommended as these tumors are of a benign nature and limited recurrences [2]. Though recurrence is rare, these patients are advised to be kept under regular follow-up, especially after a testicular sparing approach due to the limited knowledge surrounding the nature and behavior of these rare tumors.

## Conclusions

IPT are unusual tumors whose clinical presentation may resemble that of malignancy and hence careful evaluation of the lesion is essential to tailor the management individually which may vary from a testicular sparing approach to a radical orchidectomy. Though these tumors are benign in nature, due to the rarity and limited literature availability, these patients are to be advised to be kept under a periodical follow-up, especially if a testicular sparing surgery is done. Due to its rarity, we find it imperative to report this case as it can contribute important information to the existing IPT literature and might fill a gap in our understanding of this condition.
